# A clinical trial on anti-diabetic efficacy of submerged culture medium of *Ceriporia lacerata* mycelium

**DOI:** 10.1186/s12906-023-03895-z

**Published:** 2023-03-18

**Authors:** Bo-Hyung Kim, Sung-Vin Yim, Seong Deok Hwang, Yoon Soo Kim, Jeong-Hwan Kim

**Affiliations:** 1 Department of Clinical Pharmacology and Therapeutics, College of Medicine, Kyung Hee University, Seoul, 02447 Korea; 2 Bio-R&D Center, Fugenbio Co., Ltd, Seoul, 06746 Republic of Korea; 3 FugenCellTech Co Ltd, Sangju, 37272 Gyeongsangbuk-Do Korea; 4 Cardiovascular Research Institute, Graduate School of Medicine, Yokohama City University, 3-9 Fukuura, Kanazawa-Ku, Yokohama, 236-0004 Japan

**Keywords:** Type 2 diabetes, Prediabetes, Anti-diabetes, Insulin resistance, Fasting glucose tolerance, Metabolic syndrome, *Ceriporia lacerata*

## Abstract

**Background:**

Increased glucose level and insulin resistance are major factors in Type 2 diabetes mellitus (T2M), which is chronic and debilitating disease worldwide. Submerged culture medium of *Ceriporia lacerata* mycelium (CLM) is known to have glucose lowering effects and improving insulin resistance in a mouse model in our previous studies. The main purpose of this clinical trial was to evaluate the functional efficacy and safety of CLM in enrolled participants with impaired fasting blood sugar or mild T2D for 12 weeks.

**Methods:**

A total of 72 participants with impaired fasting blood sugar or mild T2D were participated in a randomized, double-blind, placebo-controlled clinical trial. All participants were randomly assigned into the CLM group or placebo group. Fasting blood glucose (FBG), HbA1c, insulin, C-peptide, HOMA-IR, and HOMA-IR by C-peptide were used to assess the anti-diabetic efficacy of CLM for 12 weeks.

**Results:**

In this study, the effectiveness of CLM on lowering the anti-diabetic indicators (C-peptide levels, insulin, and FBG) was confirmed. CLM significantly elicited anti-diabetic effects after 12 weeks of ingestion without showing any side effects in both groups of participants. After the CLM treatment, FBG levels were effectively dropped by 63.9% (ITT), while HOMA-IR level in the CLM group with FBG > 110 mg/dL showed a marked decrease by 34% up to 12 weeks. Remarkably, the effect of improving insulin resistance was significantly increased in the subgroup of participants with insulin resistance, exhibiting effective reduction at 6 weeks (42.5%) and 12 weeks (61%), without observing a recurrence or hypoglycemia. HbA1c levels were also decreased by 50% in the participants with reduced indicators (FBG, insulin, C-peptide, HOMA-IR, and HOMA-IR). Additionally, it is noteworthy that the levels of insulin and C-peptide were significantly reduced despite the CLM group with FBG > 110 mg/dL. No significant differences were detected in the other parameters (lipids, blood tests, and blood pressure) after 12 weeks.

**Conclusion:**

The submerged culture medium of CLM showed clinical efficacy in the improvement of FBG, insulin, C-peptide, HbAc1, and HOMA-index. The microbiome-based medium could benefit patients with T2D, FBG disorders, or pre-diabetes, which could guide a new therapeutic pathway in surging the global diabetes epidemic.

**Supplementary Information:**

The online version contains supplementary material available at 10.1186/s12906-023-03895-z.

## Background

As the average life expectancy has been extended due to the increase in medical benefits, multifactorial chronic metabolic disorder such as diabetes is significantly growing nowadays. There are currently about 0.5 billion people with diabetes worldwide, reaching ~ 0.7 billion by 2045, confirming the most predominated form of the leading causes of death and disabilities worldwide [1, 2]. Diabetes is characterized by insulin resistance due to chronic hyperglycemia, which is still a serious and worsening problem related to the development of diabetic complications, despite many pharmaceutical developments and global intensive efforts to control blood sugar [3]. Moreover, in the pandemic era of COVID-19, metabolic syndrome such as diabetes is rapidly increasing more than usual because of the abnormal daily living environment where exercise and eating habits are limited by non-face-to-face or quarantine measures. Recently, the long-term pandemic has increased the chances of developing diabetes: it has been reported that people with mild COVID-19 infections and no previous risk factors for diabetes also have an increased chance of developing the chronic disease [4]: People with a high body mass index (BMI), a measure of obesity, and significant risk factors for T2D more than doubled their risk of developing diabetes after infection with COVID-19.

Meanwhile, there are no clear symptoms of prediabetes, which means one may have it and not be aware until they receive testing. Over 1 billion people worldwide live with prediabetes, which is defined by increased fasting blood glucose (FBG), impaired glucose tolerance (IGT), and/or higher HbA1c. Prediabetes has an expected prevalence as that of type 2 diabetes (T2D), both of which have considered as global epidemic of the twenty-first century [5, 6]. According to the American Diabetes Association, an estimated 10 ~ 23% of people with prediabetes will move on to develop T2D within 5 years [5]. Therefore, anyone at risk of prediabetes should undergo regular checkups, including high BMI and waist circumference, age over 45, or other cardiovascular disease [5].

There is currently no fundamental cure for diabetes: although many commercial hypoglycemic drugs have been developed to control high blood sugar, they are costly with potential serious complications such as hypoglycemia, insulin resistance, and severe cardiovascular and cancer-related risks [7–10]. As a direction for finding active antidiabetic agents in natural sources, the potential of treating diabetes with edible and medicinal mushrooms combining traditional medicine and various research studies has been well demonstrated [11–13].

**Fig. 1:**
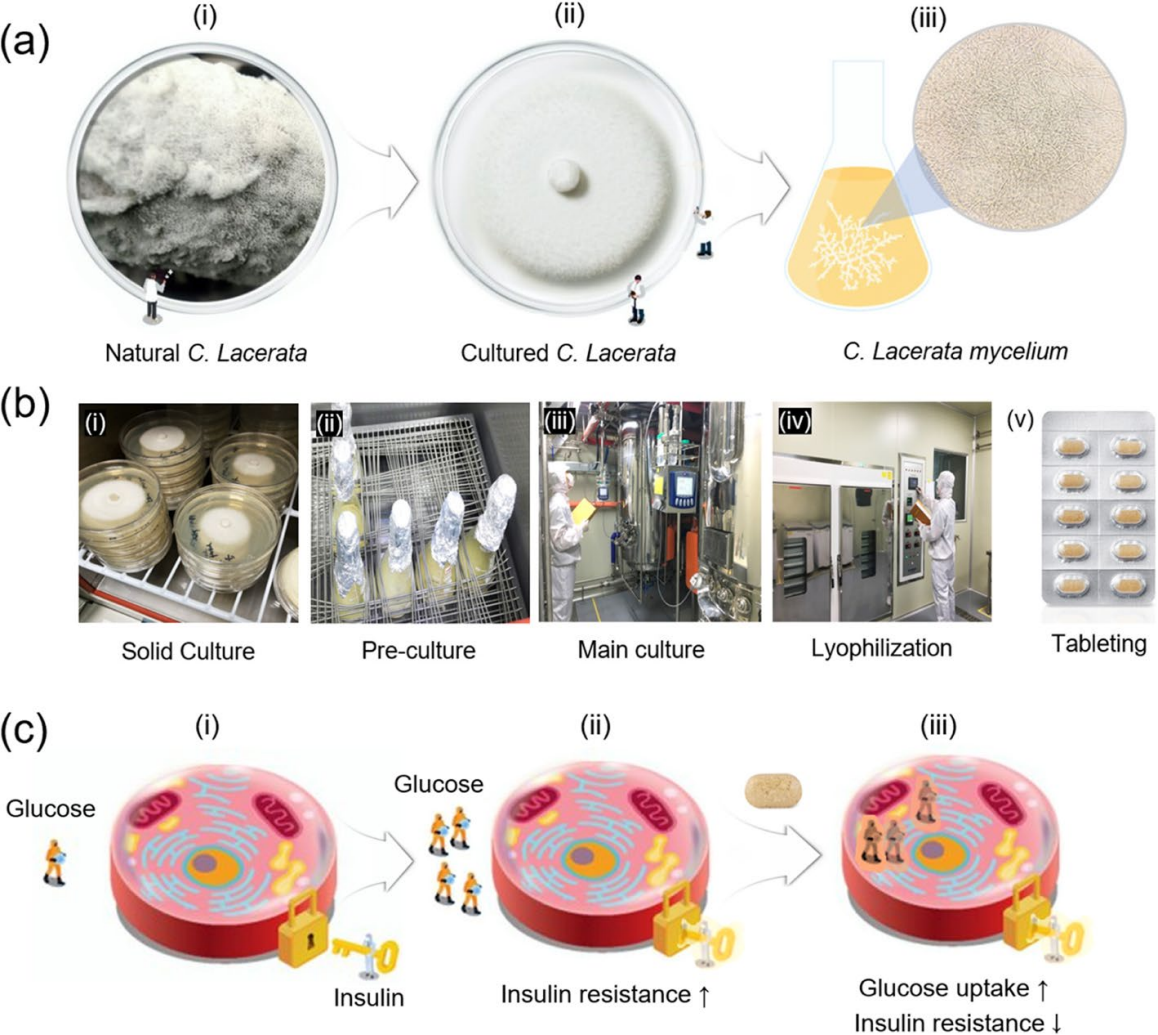
Culture procedure of submerged culture media of *C. lacerata* mycelium (**a**). The green manufacture processes of CLM tablets (**b**) including solid culture (i), pre-culture (ii), main culture (iii), lyophilization (iv), and tableting (v). A schematic anti-diabetic effect of CLM at the cellular level (**c**): normal blood glucose state (i), high blood glucose state (ii), glucose control by CLM (iii)

*Ceriporia lacerata* (*Phanerochaetaceae,* Basidiomycota) is a type of white rot fungus that decomposes cellulose and lignin of trees in the natural state to play a key role in biological reduction [14, 15] (Fig. 1a, i). The cultured *C. lacerate* is composed of microscopic polypores, so it looks like a white moss (Fig. 1a, ii). During the liquid culture process, various secondary metabolites, e.g., exo-metabolites, along with its mycelial growth, are generated depending on the environmental conditions (Fig. 1a, iii). Recently, FugenCelltech Co. Ltd has successfully developed an exclusive green mass production method of tableted culture medium of *C. lacerata* mycelium (CLM) as an anti-diabetic nutraceutical product (Cepona™) (Fig. 1b, (i)-(v)).

The submerged culture medium of CLM has been studied to control high blood glucose levels [16, 17], insulin secretion via cell protective effect [18], antihyperglycemic efficacy by lowering insulin resistance [19], and insulin signal transduction via activation of AMPK and GLUT4 [19, 20]. Therefore, it is considered that CLM has an exceptional potential as a novel functional ingredient that can help control blood sugar for people with pre-diabetes, by lowering impaired fasting blood sugar/insulin resistance, as proposed in Fig. 1c (i-iii).

In the present study, we have examined whether CLM tablet has anti-diabetic effects in Korean participants who have glucose intolerance or mild T2D. A randomized, double-blind, and placebo-controlled clinical trial was thoroughly conducted.

## Methods

### Preparation and characterization of test sample and placebo

The microbial organism used in this work was cultured after inoculation of *C. lacerata*, originally owned by FugenCellTech, Co. Ltd., Korea, in potato dextrose agar (Difco. Co., Maryland, USA) medium at 25 °C for 9 days. As a pre-culture process, 4 g/L of starch, 20 g/L of glucose, and 600 mL of purified water were mixed in the CLM liquid medium and agitated for 10 days at 300 rpm, 25 °C, pH 5, according to the prior literature [16]. After the pre-culture was completed, the mycelium culture medium was transferred to a liquid medium prepared by mixing 12.5 g/L of sucrose, 2.5 g/L of skim soybean meal, 2.5 g/L of starch, 0.125 g/L of antifoam agent, and 400 L of purified water. The culture was further incubated continuously for 9 days by injecting air at a rotational speed of 100 rpm. The submerged culture medium of CLM was freeze-dried and pulverized, and then used according to the capacity of each experimental group based on the dry weight. The raw material was manufactured without using any toxic or unauthorized chemicals and solvents at a highly controlled GMP-certified plant (acquired permission for functional nutraceutical from the Korean Ministry of Food and Drug Safety on Dec. 2019). CLM tablets were prepared using a freeze-dried culture medium of CLM. As the excipient, it is composed of crystalline cellulose, hydroxypropyl methylcellulose, silicon dioxide, and magnesium stearate. The formulation was stored at room temperature, which is not only easy to supply, but also secured its biological safety through GLP-toxicity tests such as single-dose toxicity tests (rodents), repeated-dose toxicity tests (rodents), and genotoxicity tests (return mutations, micronucleus tests, and chromosomal abnormalities tests). The placebo tablet was composed of lactose, crystalline cellulose, hydroxypropyl methylcellulose, magnesium stearate, and caramel pigments. The formulation was stored at room temperature.

### Clinical trial design and process

This study is a single-center, randomized, double-blind, placebo-controlled clinical trial. All processes were conducted at a single center (Kyung Hee University Hospital, Seoul, South Korea) from Feb. 15. 2015 (1st patients screened) to May. 5. 2015 (last patients completed). A total of 113 volunteers with impaired fasting blood sugar and mild T2D participated in the screening procedure, of which 72 participants met the inclusion and exclusion criteria and were enrolled in this study. To calculate the effective number of participants, the following assumptions were employed.

1. The statistical hypothesis test of the evaluation variable is a one-sided test.
2. Level of significance is 5%
3. Type 2 error (β) is set to 0.2 and the power of the test is maintained at 80%.
4. The ratio of the number of test samples between the test group and the control group, e.g., (Jesus of the test group) = (Jesus of the control group), 1:1.
5. After ingestion of test food, functional evaluation variables of the test group and the control group were compared.

To estimate the number of participants, the criteria for evaluating the blood glucose lowering function was used as in the human application test [21], as below:

The hypothesis is as follows:

- µt = µc (After the test, the measured value of the endpoint of the test group is the same as that of the control group)
- H1: µt < µc (After the test, the measured value of the endpoint of the test group is less than that of the control group).

Assuming the above (1)-(5), the number of test samples required for the clinical trial is as follows (one-sided test):

Formula for calculating the number of participants is expressed by the difference in the resulting variable as below,

$$\mathrm{Number}\;\mathrm{of}\;\mathrm{participants}\;(\mathrm N)=\frac{\left(\mathrm{Z}\alpha+\mathrm{Z}\beta\right)^2\ast\sigma^2\ast2}{\mathrm{E}^{2}}$$

(σ: standard deviation of the post-treatment change value in the previous trial, E: judged to be clinically significant, α = 0.05 (Zα = 1.645), β = 0.2 (Zβ = 0.840), σ = 5.4).

When the number of participants was calculated as follows by the above equation, the minimum number of participants is about 23 per group.

The effective number of samples is 23 x (120/100) x (130/100) = 35.88, when calculated by considering the 20% dropout rate and 30% compliance rate in the obtained sample number. Thus, the number of participants to be enrolled in each group is 36, and the total number of participants is 72.

The 72 selected participants were randomly assigned to the test group or control group at a ratio of 1:1 based on the following steps: (1) Patients selected as study participants were assigned to each group using a randomization method based on probability. (2) Random allocation table using a function that generates a random number of the SPSS program. (3) One of the two groups was assigned from the lowest number in the order of date of visit (Day 0). The random number (RN) was assigned according to the randomization table in the order of participation in the group to be assigned to the test group or the control group. (4) The administering pharmacist applied the medication to be administered to the participants according to the randomization plan, which was provided according to the code given to the participants. (5) The randomization of blocks using random code program of statistical program was designed to have the same number of participants.

All physicians and outcome assessors were blinded to randomization allocation. During the experiment, neither the investigator nor the participants were aware of each participant was assigned to, and they did not remove their blindfolds until a medical emergency occurred to protect the privacy of the assigned group. To ensure this, research activities, including screening, enrollment, informed consent, baseline data collection, randomization, and medication administration, were conducted solely by research personnel.

All participants were visited at the initial screening (visit 1), baseline (visit 2), 6 weeks (visit 3), 12 weeks (visit 4), and follow up period. After randomly assigning the participants to the CLM group (*n* = 36) or placebo group (*n* = 36) at a ratio of 1:1, the participants of each group were administered 2 tablets of CLM (550 mg/tablet) or 2 tablets of placebo (550 mg/tablet) before meal 3 times a day for 12 weeks. The medication compliance of the test product was measured by counting the dose or number of remaining medications at each visit. If the medication compliance was less than 80% for two consecutive times, the participants were excluded from the PP analysis.

### Clinical trial participants

Participants corresponding to all the following criteria were recruited. After the investigator fully explained the purpose and method of this test, possible risks, and rewards to the participants who wished to participate, a person who agreed to participate in this test in writing was selected as the final participant. The detail test schedule was described in Table S3. There was no change after the start of the trial designating the outcome as primary or secondary.

### Inclusion criteria

1. Participants with 20–75 years of age and non-lactating women of no childbearing potential, excluding illiterate person.
2. Participants with 100–140 mg/dL of FBG, who do not take diabetic drugs. Participants Participant with less than 7.0% of HbA1c.
3. Participants with less than 110 mg/dL of FBG even if the HbA1c is 6.5%-7.0%.
4. Participants who voluntarily decided to participate in the clinical trial after fully understanding the detailed explanation of the trial and agreed in writing to abide by these precautions.

### Exclusion criteria

1. Participants who experienced adverse reactions such as allergies when taking medicines, health functional foods, etc.
2. Participants with hypersensitivity to mushrooms or a history of the same reaction.
3. Participants with gastrointestinal diseases that may affect the absorption of the test product for human application (e. g., Crohn's disease) or a person with a history of gastrointestinal surgery (except for simple appendectomy or hernia surgery).
4. Participants who show the following results in a diagnostic medical examination.
  AST, ALT > 2 times the upper limit of the normal range.
  Other significant diagnostic medical examination findings.
5. Those who have the following clinically significant diseases; diabetes patients taking hypoglycemic drugs or insulin, patients with uncontrolled hypertension (over 140/90 mmHg), patients with blood LDL-cholesterol over 160 mg/dL, patients with thyroid dysfunction, depression, schizophrenia, alcoholism, drug addiction, heart failure, angina pectoris, cardiovascular disease, or acute and chronic liver disease (chronic hepatitis B, chronic hepatitis C, various cirrhosis, liver cancer, etc.).
6. Participants who have taken anti-obesity, anti-depressants, contraceptives, oral steroids, or female hormones. Participants who have taken merchant hormones, or who have taken drugs that affect the absorption, metabolism, and excretion of the test food, or drugs that may affect blood sugar reduction.
7. Pregnant women and lactating women.
8. Those who participated in other clinical trial within 1 month before the first intake date.
9. Participants who cannot follow the requirements of clinical trial by investigator.
10.  Participants who proved to be inappropriate by other doctors.

### Evaluation of efficacy and safety

For evaluation of anti-diabetic activity of CLM, laboratory test for FBG, HbA1c, insulin, and C-peptide were performed at each visit. Oral glucose tolerance test (OGTT) (2-h postprandial glucose test) was performed at the baseline (visits 2) and after 12 weeks (visit 4).

HOMA-IR index is the most used method for estimating insulin resistance and was calculated as follows. The product of basal glucose (mg/dL) and fasting insulin (ulU/mL) divided by 22.5. HOMA-IR by C-peptide was analyzed by replacing insulin with C-peptide in HOMA-IR formula. For safety evaluation, vital sign (systolic and diastolic blood pressure, pulse rate), electrocardiogram, laboratory test (complete blood cell count, chemistry laboratory test, urinalysis) and adverse events were thoroughly checked for participants.

### Statistical analysis

All data were statistically processed using SPSS Statistics (ver. 21.0) for Windows. Data processing for efficacy was based on an ITT analysis of participants taking the test product for which at least one primary endpoint was measured. In addition, PP analysis, which analyzes data obtained from participants who completed the study according to the human application test plan, was used as an auxiliary data. To evaluate the efficacy of the dropout participants, the analysis was performed by applying the LOCF (Last Observation Carried Forward) method, which took the final record evaluated after administration of the test product or the control product. In all tests, a statistical treatment result of *p* < 0.05 was considered significant. Less than three decimal places were not displayed.

#### Primary efficacy evaluation method

The change in FBG at the end point (6 weeks, 12 weeks (LOCF)) after administration of the test group and the control group compared to the baseline value (visit 2) was analyzed by repeated ANOVA measurement. Student's *t*-test was used to analyze the amount of change after 12 weeks compared to the baseline. In addition, ANCOVA was used to compare the mean values of study variables between the groups, after correcting the baseline value by adjusting differences in total caloric intake from differences in baseline values, at the end of the study. A *p*-value < 0.05 was considered statistically significant.

#### Secondary efficacy evaluation method

The change in the parameters (postprandial blood glucose, glycated hemoglobin (HbAlc), insulin, C-peptide, lipids) at the end of dosing compared to the baseline value was analyzed using student's *t*-test, and the change amount for 12 weeks in each group was analyzed using the paired *t*-test. In addition, ANCOVA was used to compare the mean values of study variables between the groups, after correcting the baseline value by adjusting differences in total caloric intake from differences in baseline values, at the end of the study, at the end of the study. A *p*-value < 0.05 was considered statistically significant.

Furthermore, a stratified analysis was performed based on fasting hyperglycemic participants with a FBG > 110 mg/dL on the basis that the median FBG at the baseline was 110 mg/dL. The median HOMA-IR at baseline was 1.66, which was clinically consistent with the value determined by the Japanese Diabetes Association for normal insulin resistance [22], thus sub-group analysis was performed based on HOMA-IR > 1.66 as well as HOMA-IR < 1.66. Descriptive statistics on the changes in glucose, insulin, C-peptide, HOMA-IR, and HOMA IR by C-peptide after ingestion of samples at 12 weeks compared to the baseline were achieved for each group, and the degree of change before and after the sample intake within the group was measured by paired *t*-test. Intergroup comparisons of changes at each time point were also presented to evaluate statistically significant difference by performing two-sample *t*-test or Wilcoxon rank-sum test depending on whether normality was satisfied. Analysis of HbA1c was performed by Chi-square test.

#### Safety endpoint and analysis method

For safety analysis, chi-square test was performed by identifying the number of participants with adverse reactions by group based on laboratory test items. For 12 weeks, we analyzed whether there were significant differences between the two groups in adverse reactions, laboratory test results, and vital signs between the test group and the control group.

## Results

### Patient characteristics

**Fig. 2:**
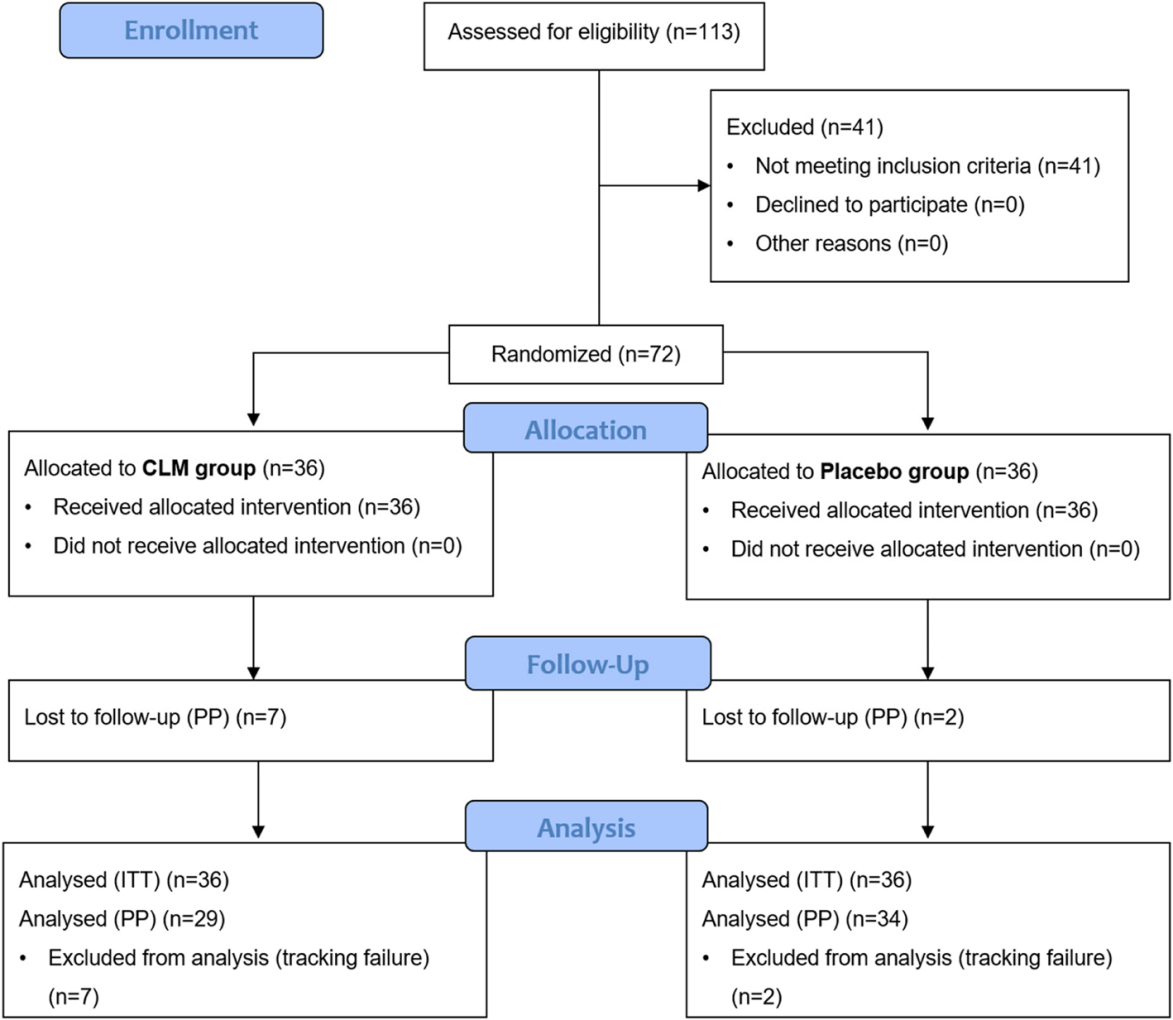
The CONSORT Diagram

Between Nov. 2013 and Jan. 2015, a total of 113 patients were screened, of which 72 were enrolled and randomized, 36 to CLM and 36 to placebo intake (Fig.**2**). Of the 72 participants, nine (7 in the CLM group and 2 in the placebo group) were dropped out due to failure to follow-up (tracking failure), and thus a total of 63 (29 in the test group and 34 in the placebo group) participants completed this clinical trial.

All participants' baseline and demographic characteristics (Table S1), progress of clinical trial protocol changes (Table S2), and the evaluation timeline (Table S3) were summarized in the Supplementary Information. Total 63 participants completed the measurements for 12 weeks without losses and exclusions. There were no significant differences between CLM and placebo group in the baseline characteristics, including age, sex, weight, height, vital sign, smoking, drinking, drugs, meals, and exercise.

### Primary efficacy analysis of CLM on fasting blood glucose (FBG)

The level of FBG as a primary endpoint was significantly decreased in CLM group (*n* = 36) after 6 weeks and 12 weeks, while that of placebo group (*n* = 36) showed no differences (Table 1).

**Table 1:** Analysis of FBG at the baseline, 6 weeks, and 12 weeks

Evaluation variable	Groups	Baseline	6 weeks	12 weeks	*p* value
Glucose (ITT)	**Placebo**	110.4 ± 8.9	111.9 ± 14.6	110.7 ± 15.5	0.504
	**CLM**	113.6 ± 9.9	106.3 ± 12.8	105.9 ± 14.0	
Glucose (PP)	**Placebo**	110.7 ± 9.1	111.8 ± 14.8	110.8 ± 16.0	0.492
	**CLM**	113.4 ± 10.3	106.8 ± 13.1	107.2 ± 15.1	

*p* values were calculated by repeated measure of ANOVA

### Effect of CLM on fasting blood glucose (FBG)

On the ITT analysis, the level of FBG as a primary endpoint was significantly decreased in CLM group after 12 weeks (7.7 ± 4.1 mg/dL, *p* = 0.034) (Table 2).

**Table 2:** Analysis of FBG from baseline to 12 weeks (ITT)

Evaluation variable		**Placebo**	**CLM**	*p* value^a^	*p* value^b^
		Mean ± SD			
Glucose	Baseline	110.4 ± 8.9	113.6 ± 9.9	0.149	
	after 12 weeks	110.7 ± 15.5	105.9 ± 14.0	0.177	
	Difference^c^	0.3 ± 6.6	-7.7 ± 4.1	0.028^*^	
	p value^d^	0.883	0.002^*^		0.034^*^

^a^Compared between groups: *p*-value by independent *t*-test

^b^Compared between groups: *p*-value by ANCOVA (adjustment with baseline and calorie)

^c^Difference between the values of baseline and after 12 weeks

^d^Compared within groups: *p*-value by paired *t*-test

^*^p < 0.05

On the PP analysis, the level of FBG as a primary endpoint was significantly decreased in CLM group after 12 weeks (6.2 ± 4.8 mg/dL, *p* = 0.045) (Table 3).

**Table 3:** Analysis of FBG from baseline to 12 weeks (PP)

Evaluation variable		**Placebo**	**CLM**	*p* value^a^	*p* value^b^
		Mean ± SD			
Glucose	Baseline	110.7 ± 9.1	113.4 ± 10.3	0.268	
	after 12 weeks	110.8 ± 16.0	107.2 ± 15.1	0.357	
	Difference^c^	0.1 ± 6.9	-6.2 ± 4.8	0.089	
	*p* value^d^	0.947	0.024^*^		0.045^*^

^a^Compared between groups: *p*-value by independent *t*-test

^b^Compared between groups: *p*-value by ANCOVA (adjustment with baseline and calorie)

^c^Difference between the values of baseline and after 12 weeks

^d^Compared within groups: *p*-value by paired *t*-test

^*^p < 0.05

### Effect of CLM on biomarkers (blood glucose, HbA1c, insulin, and C-peptide) 2 h after meal (OGTT) for 12 weeks

Two hours after intake of CLM, blood glucose, HbA1c, insulin, and C-peptide were measured (Table 4 (ITT) and Table 5 (PP)). Compared with the placebo group, the level of insulin was decreased in CLM group from 7.3 ± 5.5 to 5.0 ± 4.9 uIU/mL in ITT analysis (*p* = 0.062); from 7.0 ± 5.5 to 5.0 ± 4.9 uIU/mL in PP analysis (*p* = 0.226), although the change in insulin did not show statistical significance. Particularly, the level of C-peptide was noticeably reduced in CLM group (*p* = 0.015 (ITT), *p* = 0.025 (PP)).

**Table 4:** Analysis of OGTT from baseline to 12 weeks (ITT)

		**Placebo**	**CLM**	*p* value^a^	*p* value^b^
		Mean ± SD			
Blood glucose	Baseline	148.0 ± 47.3	150.4 ± 58.6	0.848	
	after 12 weeks	138.5 ± 57.3	140.8 ± 51.6	0.862	
	Difference^c^	-9.5 ± 10.0	-9.6 ± 7.0	0.990	
	p value^d^	0.390	0.186		0.948
HbA1c	Baseline	5.7 ± 0.6	5.8 ± 0.5	0.519	
	after 12 weeks	5.8 ± 0.5	5.8 ± 0.5	0.987	
	Difference^c^	-0.1 ± 0.1	0.0 ± 0.0	0.274	
	p value^d^	0.891	0.126		0.324
Insulin	Baseline	6.6 ± 4.8	7.3 ± 5.5	0.599	
	after 12 weeks	7.2 ± 7.8	5.0 ± 4.9	0.149	
	Difference^c^	-0.6 ± 3.0	-2.3 ± 0.6	0.090	
	p value^d^	0.604	0.062		0.137
C-peptide	Baseline	2.2 ± 1.0	2.4 ± 1.4	0.542	
	after 12 weeks	2.2 ± 1.4	1.7 ± 0.7	0.048	
	Difference^c^	0.0 ± 0.4	-0.7 ± 0.7	0.014^*^	0.015^*^
	p value^d^	0.729	0.002^*^		

^a^Compared between groups: *p*-value by independent *t*-test

^b^Compared between groups: *p*-value by ANCOVA (adjustment with baseline and calorie)

^c^Difference between the values of baseline and after 12 weeks

^d^Compared within groups: *p*-value by paired *t*-test

^*^p < 0.05

**Table 5:** Analysis of OGTT from baseline to 12 weeks (PP)

		Placebo	CLM	*p* value^a^	*p* value^b^
		Mean ± SD			
Blood glucose	Baseline	147.4 ± 48.4	148.3 ± 56.6	0.945	
	after 12 weeks	138.4 ± 59.0	143.4 ± 56.8	0.737	
	Difference^c^	-9.0 ± 67.1	-4.9 ± 0.2	0.775	
	p value^d^	0.444	0.484		0.751
HbA1c	Baseline	5.7 ± 0.6	5.8 ± 0.5	0.638	
	after 12 weeks	5.8 ± 0.5	5.8 ± 0.6	0.976	
	Difference^c^	0.1 ± 0.1	0.0 ± 0.1	0.417	
	p value^d^	0.711	0.358		0.502
Insulin	Baseline	6.8 ± 4.8	7.0 ± 5.5	0.870	
	after 12 weeks	7.3 ± 8.0	5.3 ± 5.3	0.269	
	Difference^c^	0.5 ± 3.2	-1.7 ± 0.2	0.243	
	p value^d^	0.686	0.226		0.172
C-peptide	Baseline	2.2 ± 1.0	2.2 ± 1.0	0.839	
	after 12 weeks	2.2 ± 1.4	1.8 ± 0.7	0.104	
	Difference^c^	0.0 ± 0.4	-0.4 ± 0.3	0.074	0.025*
	p value^d^	0.898	0.010*		

^a^Compared between groups: *p*-value by independent *t*-test

^b^Compared between groups: *p*-value by ANCOVA (adjustment with baseline and calorie)

^c^Difference between the values of baseline and after 12 weeks

^d^Compared within groups: *p*-value by paired *t*-test

^*^p < 0.05

### Stratification analysis of FBG, insulin, C-peptide, HOMA-IR, and HOMA-IR by C-peptide from baseline to 12 weeks (FBG ≥ 110 mg/dl)

To clarify the antidiabetic efficacy of CLM in participants with hyperglycemia, stratified analyses (ITT and PP) were performed based on a median FBG value of 110 mg/dl, as CLM was supposed to be more effective in the group with hyperglycemia (FBG ≥ 110 (*N* = 36)). The results of setting the FBG level of 110 mg/dl as the stratified value were dramatic: all the parameters (FBG, insulin, C-peptide, HOMA-IR, and HOMA-IR by C-peptide) showed significant changes in both ITT and PP analyses, providing a better window for the index changes, compared with the OGTT results (Tables 6 and 7). Moreover, these results implied that CLM is more efficacious in hyperglycemic participants.

**Table 6:** Analysis of changes in glucose, insulin, C-peptide, HOMA-IR, and HOMA-IR by C-peptide from baseline to 12 weeks (ITT)

		Placebo (*N* = 18)	CLM (*N* = 20)	*p* value
		Mean ± SD		
Blood glucose	Baseline	117.00 ± 7.85	120.55 ± 7.83	0.1680^&^
	after 6 weeks	116.78 ± 16.18	111.75 ± 19.11	
	Difference	-0.22 ± 16.30	-8.80 ± 18.25	0.1369^*^
	*p*-value^**^	0.9546	0.0441	
	after 12 weeks	117.83 ± 18.29	109.90 ± 21.02	
	Difference	0.83 ± 16.19	-10.65 ± 18.94	0.0158^&^
	*p*-value^**^	0.8297	0.0211	
Insulin	Baseline	8.37 ± 3.80	9.72 ± 5.12	0.3656^*^
	after 6 weeks	7.92 ± 5.17	6.75 ± 5.56	
	Difference	-0.45 ± 6.21	-2.97 ± 5.20	0.1818^*^
	*p*-value^**^	0.7622	0.0193	
	after 12 weeks	9.75 ± 10.08	4.65 ± 3.68	
	Difference	1.38 ± 8.86	-5.08 ± 4.94	0.0114^&^
	*p*-value^**^	0.5166	0.0002	
C-peptide	Baseline	2.53 ± 1.01	2.91 ± 1.58	0.5484^&^
	after 6 weeks	2.31 ± 1.03	2.16 ± 0.93	
	Difference	-0.22 ± 0.94	-0.75 ± 1.39	0.3883^&^
	*p*-value^**^	0.3428	0.0264	
	after 12 weeks	2.58 ± 1.78	1.87 ± 0.77	
	Difference	0.05 ± 1.13	-1.04 ± 1.63	0.0178^&^
	*p*-value^**^	0.8366	0.0099	
HOMA-IR	Baseline	2.44 ± 1.19	2.87 ± 1.50	0.3423^*^
	after 6 weeks	2.32 ± 1.84	1.89 ± 1.60	
	Difference	-0.13 ± 2.18	-0.98 ± 1.59	0.1715^*^
	*p*-value^**^	0.8075	0.0121	
	after 12 weeks	3.14 ± 3.77	1.29 ± 1.06	
	Difference	0.69 ± 3.36	-1.59 ± 1.62	0.0089^&^
	*p*-value^**^	0.3940	0.0003	
HOMA-IR by C-peptide	Baseline	4.00 ± 1.82	4.64 ± 2.46	0.3420^&^
	after 6 weeks	3.72 ± 2.1	3.29 ± 1.63	
	Difference	-0.28 ± 2.01	-1.35 ± 2.38	0.2364^&^
	*p*-value^**^	0.5647	0.0201	
	after 12 weeks	4.35 ± 3.76	2.83 ± 1.55	
	Difference	0.35 ± 2.41	-1.81 ± 2.85	0.0105^&^
	*p*-value^**^	0.5425	0.0103	

^*^Compared between groups; *p*-value by two sample *t*-test

^&^Compared between groups; *p*-value by Wilcoxon rank sum test

^**^Compared within groups; *p*-value by paired *t*-test

**Table 7:** Analysis of changes in glucose, insulin, C-peptide, HOMA-IR, and HOMA-IR by C-peptide from baseline to 12 weeks (PP)

		Placebo (*N* = 18)	CLM (*N* = 14)	*p* value
		Mean ± SD		
Blood glucose	Baseline	117.00 ± 7.85	122.14 ± 7.94	0.0702^&^
	after 6 weeks	116.78 ± 16.1	110.07 ± 16.85	
	Difference	-0.22 ± 16.30	-12.07 ± 15.00	0.0432^*^
	*p*-value^**^	0.9546	0.0100	
	after 12 weeks	117.83 ± 18.29	108.86 ± 19.02	
	Difference	0.83 ± 16.19	-13.29 ± 15.74	0.0191^*^
	*p*-value^**^	0.8297	0.0076	
Insulin	Baseline	8.37 ± 3.80	10.16 ± 5.35	0.2768^*^
	after 6 weeks	7.92 ± 5.17	8.05 ± 6.17	
	Difference	-0.45 ± 6.21	-2.11 ± 5.27	0.4032^&^
	*p*-value^**^	0.7622	0.1582	
	after 12 weeks	9.75 ± 10.08	5.41 ± 3.98	0.0238^&^
	Difference	1.38 ± 8.86	-4.74 ± 4.87	
	*p*-value^**^	0.5166	0.0030	
C-peptide	Baseline	2.53 ± 1.01	2.61 ± 1.05	0.8150^*^
	after 6 weeks	2.31 ± 1.03	2.24 ± 1.01	
	Difference	-0.22 ± 0.94	-0.37 ± 0.81	0.6273^*^
	*p*-value^**^	0.3428	0.1080	
	after 12 weeks	2.58 ± 1.78	1.98 ± 0.72	
	Difference	0.05 ± 1.13	-0.63 ± 0.95	0.0752^*^
	*p*-value^**^	0.8366	0.0263	
HOMA- IR	Baseline	2.44 ± 1.19	3.03 ± 1.57	0.2375^*^
	after 6 weeks	2.32 ± 1.84	2.25 ± 1.79	
	Difference	-0.13 ± 2.18	-0.78 ± 1.65	0.3568^*^
	*p*-value^**^	0.8075	0.0983	
	after 12 weeks	3.14 ± 3.77	1.49 ± 1.17	
	Difference	0.69 ± 3.36	-1.54 ± 1.66	0.0159^&^
	*p*-value^**^	0.3940	0.0042	
HOMA-IR by C-peptide	Baseline	4.00 ± 1.82	4.22 ± 1.59	0.5062^&^
	after 6 weeks	3.72 ± 2.16	3.33 ± 1.61	
	Difference	-0.28 ± 2.01	-0.89 ± 1.56	0.3543^*^
	*p*-value^**^	0.5647	0.0520	
	after 12 weeks	4.35 ± 3.76	2.92 ± 1.32	
	Difference	0.35 ± 2.41	-1.31 ± 1.90	0.0289^&^
	*p*-value^**^	0.5425	0.0232	

^*^Compared between groups; *p*-value by two sample *t*-test

^&^Compared between groups; *p*-value by Wilcoxon rank sum test

^**^Compared within groups; *p*-value by paired *t*-test

**Fig. 3:**
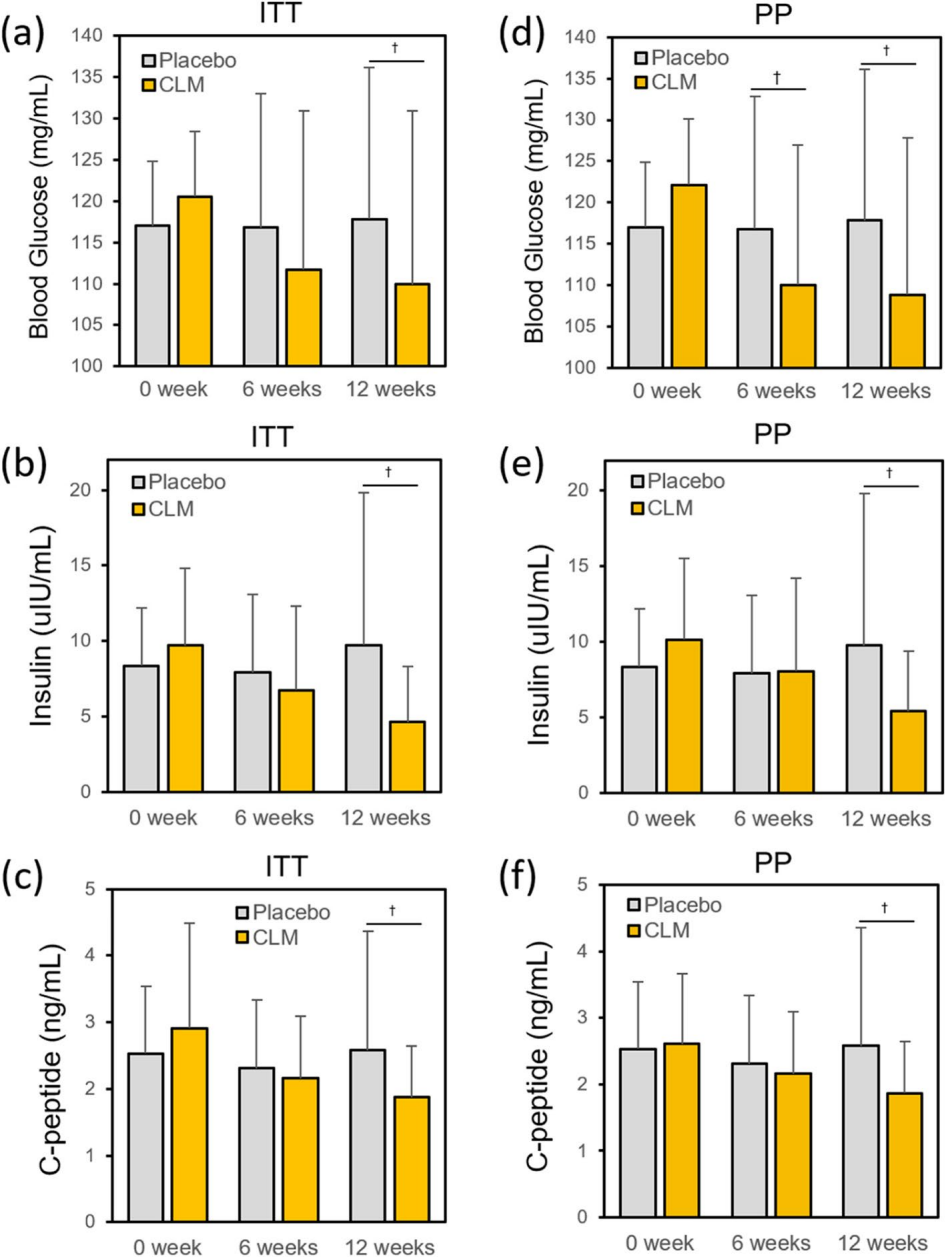
Effect of CLM on FBG, insulin, and C-peptide († *p* < 0.05)

Subsequently, the effectiveness of CLM on lowering all the anti-diabetic indicators (insulin, C-peptide, FBG levels) was validated (Fig. 3, Tables 6 and 7**)**. Interestingly, after 6 weeks, the FBG level in the CLM-ingested group was significantly reduced (7.3% (ITT); 9.9% (PP)) even though the levels of fasting blood insulin (30.6% (ITT); 20.77% (PP)) and C-peptide (25.8% (ITT); 17.2% (PP)) were significantly decreased. After 12 weeks, the levels of all the parameters were further dropped: the FBG level in the CLM-ingested group was significantly reduced (8.8% (ITT); 10.9% (PP)) even though the levels of fasting blood insulin (52.2% (ITT); 46.8% (PP)) and C-peptide (35.7% (ITT); 28.4% (PP)) were decreased. On the other hand, there was no change in all parameters in the placebo group. Moreover, it can be inferred that the result of decreased FBG despite dropped level of insulin (Fig. 3) is due to decreased insulin resistance by CLM.

**Fig. 4:**
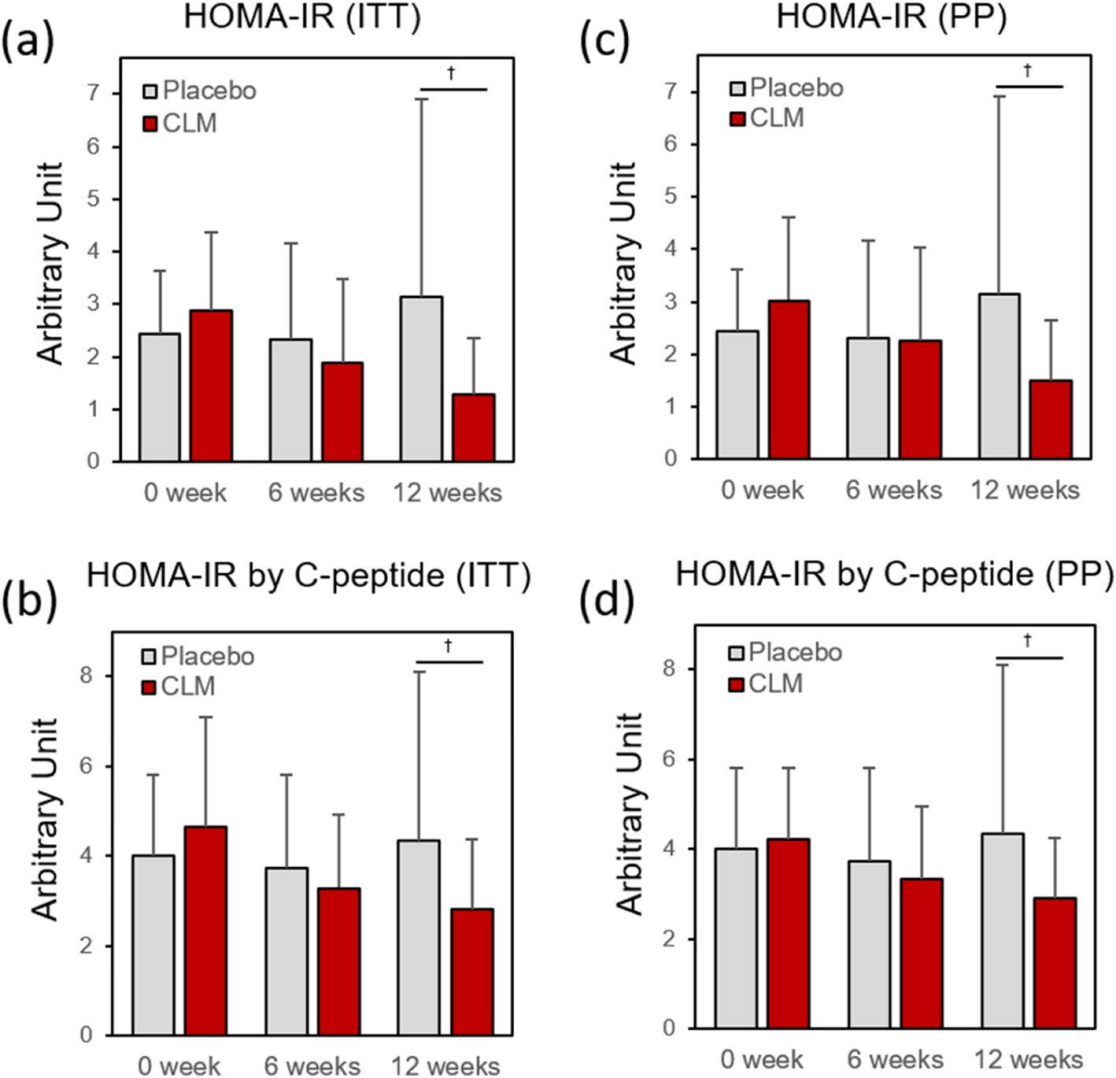
Effect of CLM on HOMA-IR and HOMA-IR by C-peptide († *p* < 0.05)

HOMA-IR and HOMA-IR by C-peptide analyses were performed to confirm the effect of CLM on insulin resistance and glucose utilization efficacy as presented in Fig. 4. While the HOMA-IR level showed an increasing trend in the placebo group, the CLM group showed a marked decrease by 34.2% at 6 weeks, 55.1% at 12 weeks (ITT) (25.8% at 6 weeks, 50.8% at 12 weeks (PP). For the HOMA-IR by C-peptide, that of the CLM group showed a marked decrease by 29.1% at 6 weeks, 39.0% at 12 weeks (ITT) (21.1% at 6 weeks, 30.8% at 12 weeks (PP)), whereas it was unchanged in the placebo group.

### Stratification analysis of HOMA-IR in participant with insulin resistance and normal participant

**Fig. 5:**
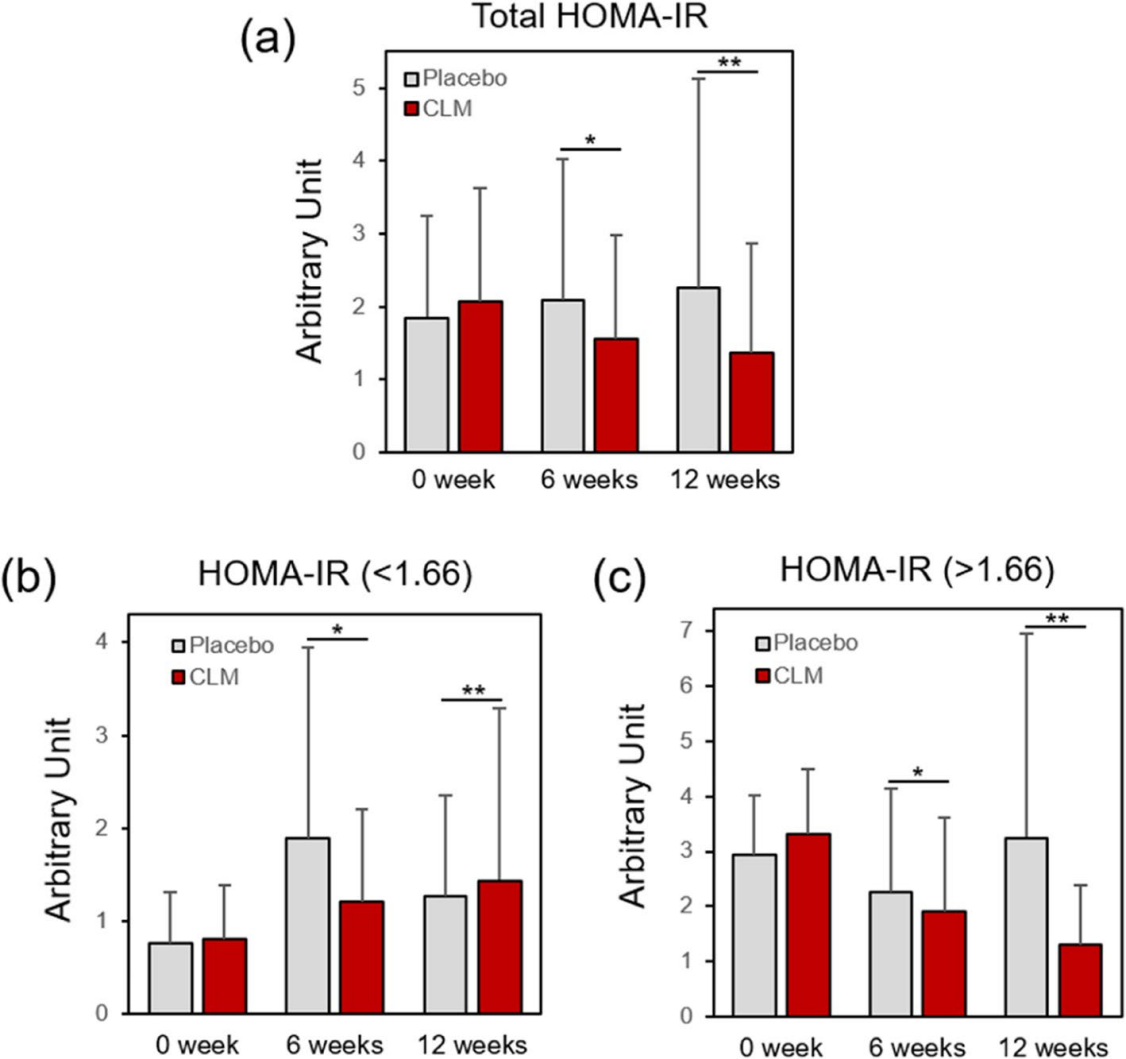
Effect of CLM against insulin resistance (**a-c**), exhibiting that the total HOMA-IR in the CLM group was substantially reduced at 6 weeks (*p = 0.046) and 12 weeks (**p = 0.018), while the placebo group was slightly increased (**a**). While maintaining normal HOMA-IR range in the participants whose insulin resistance was normal (initial HOMA < 1.66) through 6 weeks (*p = 0.5344) and 12 weeks (**p = 0.438) (**b**), a dramatic reduction was observed in the CLM group (initial HOMA-IR > 1.66) at 6 weeks (*p = 0.026) and 12 weeks (**p = 0.007) (**c**)

The effectiveness of CLM on improving insulin resistance against participant with insulin resistance (HOMA-IR > 1.66) was validated compared with normal participant (HOMA-IR < 1.66) as shown in Fig. 5. While the HOMA-IR level showed an increasing trend in the placebo group, the CLM group in the entire participant group showed a marked decrease by 34% up to 12 weeks (Fig. 5a). In the normal group with low insulin resistance, it was revealed that HOMA-IR level remained within the normal range except for a sudden increase in HOMA-IR level in the placebo group after 6 weeks (Fig. 5b). Remarkably, in the group showing insulin resistance, it was dropped up to 6 weeks in the placebo group and then gradually increased again at 12 weeks, whereas in the CML group, it significantly reduced at both 6 weeks (42.5%) and 12 weeks (61%), without showing a recurrence (Fig. 5c).

Further statistical analysis revealed that participants with decreased HbA1c with reduced markers (FBG, insulin, C-peptide, HOMA-IR, HOMA-IR) were more distributed in the CLM intake group (50%) than the placebo group (36.1%) (Table S4). In particular, the participants with lowered FBG and HbA1c were dominated in the CLM group (47.6%) compared to the placebo group (12.5%). Therefore, it is presumed that CLM lowered FBG and relatively decreased glycated hemoglobin.

### Effect of CLM on lipids

In lipid analysis, there was no significant differences in each group before and after 12 weeks of testing, and there was no difference in the change value (Table S5).

### Safety evaluation

#### Symptoms of adverse reaction

Of the 72 participants receiving the human test product, a total of 7 adverse reactions occurred in 6 participants during the test period. The adverse reactions that occurred were pruritus in 1 patient, diarrhea and digestive disorder in 1 patient, digestive disorder in 1 patient, *Helicobacter pyrori* infection in 1 patient, tailbone pain in 1 patient, and digestive disorder in 1 patient. These adverse events were not related with the CLM or placebo samples. During the whole human trial period, there were no abnormal findings on the physical examination of any participants, and no serious adverse events occurred. Therefore, CLM is considered a safe ingredient/nutraceutical with no clinically significant adverse reactions even after 12 weeks of use. Consequently, in patients with impaired fasting glucose or mild T2D, 12 weeks of CLM intake could reduce fasting glucose and C-peptide without clinically significant or adverse side effects.

#### Changes in laboratory test results

Changes in laboratory test results were analyzed using a paired *t*-test for change at the end point compared to the baseline value. There were no statistically significant changes before and after the test in both groups in vital signs (Table S6), general blood test (Table S7), and blood biochemical test (Table S8).

## Discussion

In our 12 weeks trial carried out on participants with impaired FBG or mild T2D, we observed that CLM was able to effectively improve the serum levels of FBG, insulin, C-peptide, and HOMA index, with an optimal tolerability profile that is important to guarantee long-term compliance of the treatment on prediabetes or T2D.

In OGTT analyses (ITT), the levels of FBG, HbA1c, insulin, and C-peptide were decreased by 6.38%, 0%, 31.5%, and 29.2% (*p* < 0.05), respectively (in PP, 3.31%, 0%, 24.3%, and 18.2% (*p* < 0.05)). In stratification analyses at FBG ≥ 110 mg/dl (ITT), the levels of FBG, insulin, C-peptide, HOMA-IR, and HOMA-IR by C-peptide were significantly reduced (*p* < 0.05) by 8.8%, 52.2%, 35.7%, 55.1%, and 39%, respectively (in PP, 10.9%, 46.8%, 28.4%, 50.8%, and 30.8%). This is presumed to be the result of reduced FBG despite lowered insulin levels due to reduced insulin resistance by CLM. Based on the HOMA-IR index > 1.66, the effect of improving insulin resistance was remarkably increased in the subgroup of participants with insulin resistance, exhibiting effective reduction at 6 weeks (42.5%) and 12 weeks (61%), without observing a recurrence or hypoglycemia. HbA1c levels were also decreased by 50% in the participants with reduced indicators.

Usually, it is known that insulin level increases due to the rise of c-peptide when administering functional foods or hypoglycemic agents, thus there is a periodic mechanism in which blood sugar decreases. Therefore, it was found that the hypoglycemic effect of CLM decreased blood sugar, leading to reduce insulin demand along with decrease of C-peptide, a precursor of insulin, accordingly.

Therefore, the effect of CLM on improving insulin resistance was statistically proved through the stratified analysis, which confirmed that the hypoglycemic effect in all the CLM-ingested groups was possibly due to the decrease in insulin resistance. It is expected that oral administration of CLM would benefit patients with mild T2D, pre-diabetes, glucose tolerance, impaired FBG, or insulin resistance, since it has been demonstrated that there is an obvious improvement of all parameters in participants with insulin resistance.

Impaired FBG (IFG) and impaired glucose tolerance (IGT) are pre-diabetes stage associated with insulin resistance [23]. Recent studies including intensive lifestyle interventions [24] and meta-analyses [23, 25] have shown that physical activity and diet can reduce FBG levels and prevent or delay the transition between IFG, IGT and T2DM. CLM showed efficient anti-diabetic effect without clinically significant adverse side effects or recurrence for 12 months in patients with IFG, IGT, or T2DM, providing evidence for the health benefits of 12 weeks of CLM treatment in patients who do not reach their goal glycemia despite continuous medications such as metformin, sulfonylurea, DPP-4 inhibitors, and so on.

The anti-diabetic efficacy was consistent with our previous study using high fat diet diabetic mouse model [18]: it was observed that levels of FBG, blood insulin, and C-peptide were significantly decreased in the CLM group and Metformin compared to a control group, and the effect of improving insulin resistance and reducing FBG was equivalent to Metformin. Combining both human and animal data, it becomes noteworthy that CLM visibly reduces FBG despite a decrease in blood insulin, due to improved insulin resistance.

When CLM was immersed in culture for 10 days, the submerged culture medium contained mycelium (5.2 g/L) and EPS (1.62 g/L). In particular, the β-glucan content was about 15.69% (w/w) [16]. It has been studied that the major bioactive components of CLM included EPS composed of mannose (83.36%), galactose (12.54%), and glucose (4.10%) [26], as well as some flavonoids and sesquiterpenoids [27, 28]. Furthermore, the CLM manufacturing process is not an extraction process that mainly uses chemical organic solvents, but a bio-/eco-friendly, green microbial culture process, which can be well fit to the latest green manufacturing trends in the market.

In most diabetes-related clinical trials, it is difficult to manage the response to drugs or test samples due to differences in blood glucose levels of individual diabetic patients. In other words, if functional foods/supplements tailored to individual pharmacological characteristics and improved blood sugar control management methods for clinical participants can be combined, an era in which so-called antidiabetics can be personalized to everyone can be expected. In this context, despite the numerous antidiabetic drugs currently on the market and the introduction of various new treatments in the future, exercise, and diet control along with a steady intake of appropriate functional supplements tailored to each individual are eventually fundamental in the prevention and treatment of diabetes. In this regard, although this study is a clinical study limited to Korea, the use of CLM as a promising solution for prediabetes and diabetes could be widely applied worldwide to address the global diabetes epidemic.

Further studies are needed to elucidate the pharmacological potential and molecular mechanisms of cellular properties of active compounds derived from *C. lacerata*. In the future, if the biological activity and stability could be secured by identifying the active compound of the CLM component and conducting additional clinical trials, it would be developed as a new anti-diabetic therapeutic agent in the future. Still, because of the potential that microbial-derived natural culture medium components can appreciably improve or protect glycemic status against T2D, they can be considered as a niche opening their application as antidiabetic nutraceuticals/medical foods.

## Conclusions

CLM as a novel microbial ingredient elicited substantially positive anti-diabetic effects during the intake period (12 weeks), involving especially ameliorated glycemic control of FBG, peripheral insulin action, and reduced C-peptide and HbA1c, via improving insulin resistance. CLM is also considered a safe ingredient with no adverse reactions after 12 weeks of use. To the best of our knowledge, this is a well-defined human randomized controlled trial to examine the anti-diabetic effect of novel microbial mycelium on hypoglycemia and insulin resistance. The results of this study proposed that the microbial medium of CLM might reduce blood sugar, especially FBG and insulin tolerance, and its effect could be helpful for patients with impaired FBG or pre-diabetes. The efficacy of CLM against T2D could be beneficial as a functional supplementation considering that the size of the population with metabolic disorder (T2D, prediabetes, obesity) has been increasing, while anti-diabetic treatment is limited.

## Supplementary Information

**Additional file 1: Table S1. **Demographic baseline characteristics of subjects. **Table S2. **Progress of changes to the clinical trial protocol. **Table S3. **Schedule of clinical study assessments. **Table S4. **Effectof CLM on lowering HbA1c over subjects with decreased levels of FBG, insulin, C-peptide, insulin, HOMA-IR, and HOMA-IR by C-peptide. **Table S5. **Lipid changes at the baseline and after 12 weeks. **Table S6 **Changes in vital signs. **Table S7. **Changes in stability evaluation index (blood test). **Table S8. **Changes in stability evaluation index (biochemical test). 

## Data Availability

The datasets used or analyzed during the study are available from the corresponding author on reasonable request.

## Abbreviations

ALP: Alkaline phosphatase
ALT L: Alanine aminotransferase
GPT: Glutamic-pyruvic transaminase
FBG: Fasting blood glucose
BUN: Blood urea nitrogen
BMI: Body mass index
CLM: *Ceriporia lacerata* Mycelium
ECG: Electrocardiogram
HbA1c: Plasma Hemoglobin A1c
HDL: High density lipoprotein
HOMA-IR: Homeostasis model assessment for insulin resistance
GMP: Good manufacture practice
IRB: Institutional Review Board
ITT: Intention To Treat
LDL: Low density lipoprotein
OGTT: Oral glucose tolerance test
PP: Per Protocol
TG: Triglyceride
T2D: Type 2 diabetes
