# Supplementary material for: A clinical trial on anti-diabetic efficacy of submerged culture medium of Ceriporia lacerata mycelium
Source: BMC Complement Med Ther. 2023 Mar 18;23:83. doi: 10.1186/s12906-023-03895-z (PMC10024018; doi:10.1186/s12906-023-03895-z)
Supplement: Supplementary file 1 — Additional file 1: Table S1. Demographic baseline characteristics of subjects. Table S2. Progress of changes to the clinical trial protocol. Table S3. Schedule of clinical study assessments. Table S4. Effectof CLM on lowering HbA1c over subjects with decreased levels of FBG, insulin, C-peptide, insulin, HOMA-IR, and HOMA-IR by C-peptide. Table S5. Lipid changes at the baseline and after 12 weeks. Table S6 Changes in vital signs. Table S7. Changes in stability evaluation index (blood test). Table S8. Changes in stability evaluation index (biochemical test). [file 12906_2023_3895_MOESM1_ESM.docx]

***Supplementary Information***

**A clinical trial on anti-diabetic efficacy of submerged culture medium of *Ceriporia lacerata* mycelium**

**Table S1.** Demographic baseline characteristics of subjects

| Evaluation variables | **Placebo (n=36)** | **CLM (n=36)** | *p* value |
| --- | --- | --- | --- |
| Age (years) | 57.97±7.28 | 58.08±8.23 | 0.952^a^ |
| Gender (n,%) |  |  |  |
| Man | 18 (50.0) | 18 (50.0) | 1.000^b^ |
| Woman | 18 (50.0) | 18 (50.0) |  |
| Weight (kg) | 66.79±10.80 | 64.97±10.26 | 0.467^a^ |
| Height (cm) | 161.19±8.76 | 161.81±9.05 | 0.772^a^ |
| Systolic blood pressure (mmHg) | 127.08±9.20 | 123.44±11.17 | 0.136^a^ |
| Diastolic blood pressure (mmHg) | 77.69±7.84 | 75.44±8.80 | 0.256^a^ |
| Pulse (beat/min.) | 70.94±10.16 | 73.28±11.28 | 0.359^a^ |
| Smoking history (n,%) |  |  |  |
| Non-smoker | 21 (58.3) | 23 (65.7) | 0.194^b^ |
| Past smoker | 10 (27.8) | 4 (11.4) |  |
| Current smoker | 5 (13.9) | 8 (22.9) |  |
| Drinking history (n,%) |  |  |  |
| No | 10 (27.8) | 13 (36.1) | 0.834^b^ |
| Small alcoholic beverages (<½ bottle of soju/week) | 14 (38.9) | 12 (33.3) |  |
| Large amount of alcohol (>½ bottle of soju/week) | 12 (33.3) | 11 (30.6) |  |
| Concomitant drugs (n,%) |  |  |  |
| None | 18 (50.0) | 18 (50.0) | 1.000^b^ |
| Has exist | 18 (50.0) | 18 (50.0) |  |
| Medical history and accompanying diseases |  |  |  |
| None | 16 (44.4) | 16 (38.9) | 0.811^b^ |
| Has exist | 20 (55.6) | 22 (66.1) |  |
| Meal |  |  |  |
| Regular | 32 (88.9) | 30 (83.3) | 0.735^b^ |
| Irregular | 4 (11.1) | 6 (16.7) |  |
| Exercise |  |  |  |
| Not at all | 11 (30.6) | 10 (27.8) | 0.745^b^ |
| Irregular (30 min, less than 3 times a week) | 6 (16.7) | 9 (25.0) |  |
| Regular (30 min, 3 times a week or more) | 19 (52.8) | 17 (47.2) |  |

^a^ *p* value by independent *t*-test

^b^ *p* value by Chi-square test

**Table S2.** Progress of changes to the clinical trial protocol

| Division | Order | Plan | Approval date | Contents |  | |  |
| --- | --- | --- | --- | --- | --- | --- | --- |
|  |  | Version |  |  |  | |  |
|  |  |  |  |  |  | |  |
|  |  |  |  | IRB |  |  |  |
|  | 1 | 1.0 | 2013.09.16 | First sign of clinical trial protocol |  |  |  |
|  |  |  |  |  |  |  |  |
|  |  |  |  |  |  |  |  |
|  | 2 | 1.0 | 2013.10.01 | Automatic Test Subject Ver1.1 |  |  |  |
|  |  |  |  | Recruitment Notice Ver1.1 |  |  |  |
|  |  |  |  |  |  |  |  |
|  |  |  |  | Test Plan Ver1.2 |  |  |  |
|  |  |  |  |  |  |  |  |
|  |  |  |  | Case Report Ver1.2 |  |  |  |
|  |  |  |  | Automatic Test Subject Ver1.2 |  |  |  |
|  |  |  |  | Recruitment Notice Ver1.2 |  |  |  |
|  |  |  |  |  |  |  |  |
|  | 3 | 1.2 | 2013.11.06 | Recruitment Flyer Ver1.2 |  |  |  |
|  |  |  |  |  |  |  |  |
|  |  |  |  | Signature page Ver1.2 |  |  |  |
|  |  |  |  |  |  | |  |
|  |  |  |  | Research Fund Ver1.1 |  |  |  |
| Kyunghee University Hospital |  |  |  | Research Fund Ver1.2 |  |  |  |
|  |  |  |  | Case Report Ver1.2 |  |  |  |
|  | 4 | 1.2 | 2013.11.18 | Case Report Ver1.3 |  |  |  |
|  |  |  |  |  |  |  |  |
|  |  |  |  |  |  |  |  |
|  |  |  |  |  |  | |  |
|  | 5 | 1.2 | 2014.02.24 | Recruitment Notice Ver1.3 |  |  |  |
|  |  |  |  |  |  |  |  |
|  |  |  |  |  |  |  |  |
|  |  |  |  |  |  | |  |
|  |  |  |  |  |  |  |  |
|  |  |  |  |  |  |  |  |
|  |  |  |  |  |  |  |  |
|  |  | 1.2 | 2014.06.14 | Test Plan Ver1.2 |  |  |  |
|  | 6 |  |  |  |  |  |  |
|  |  |  |  |  |  |  |  |
|  |  |  |  |  |  |  |  |
|  |  |  |  |  |  |  |  |
|  |  |  |  |  |  |  |  |
|  |  |  |  |  |  | |  |

**Table S3.** Schedule of clinical study assessments.

|  |  | Screening | Administration period | | | | |  |
| --- | --- | --- | --- | --- | --- | --- | --- | --- |
|  |  | Visit | 1 | 2 | 3 | 4 |  |  |
|  |  | Week | - 2 | 0 | 6 | 12 | Follow up^6)^ |  |
|  | Window period | |  | 0 | ± 7 | ± 7 |  |  |
|  | Written consent | | O |  |  |  |  |  |
| Demographic Survey (gender, date of birth, age) | | | O |  |  |  |  |  |
|  | Physical examination^1)^ | | O |  |  | O |  |  |
|  | Living habit survey | | O | O | O | O |  |  |
| Investigation of medical history and drug use history | | | O | O | O | O | O |  |
|  | Dietary Survey^2)^ | |  |  | O | O |  |  |
|  |  | Adverse reaction monitoring |  |  | O | O | O |  |
| Safety |  | Vital signs | O | O | O | O | O |  |
| evaluation^3)^ |  | Cardiography |  |  |  | O | O |  |
|  |  | Diagnostic medical examination | O |  | O | O | O |  |
|  |  | Fasting blood sugar | O | O | O | O |  |  |
| Functional |  | Glycemic hemoglobin (HbA1c) | O | O | O | O |  |  |
| evaluation^4)^ |  | Insulin, C-peptide | O | O | O | O |  |  |
|  |  | After meal 2 hours blood sugar |  | O |  | O |  |  |
|  | Concomitant assessment^5)^ | |  |  |  |  |  |  |
| Randomization | | |  | O |  |  |  |  |
| Distribution of test foods and reference foods | | |  | O | O |  |  |  |
| Returned food collection | | |  |  | O | O |  |  |
| Confirmation of changes in drug use | | |  | O | O | O |  |  |

1) Physical examination: During the test period, the same scale was used, and the height was measured in cm after taking off shoes at the first visit. However, only body weight was measured at visit 4.

2) Dietary survey: All foods consumed from 0 o'clock to midnight on the day before the survey were investigated by a 24 h recall method. Using this data, the intake of the food group and the food group per day of the survey subject was calculated, and the amount of nutrient intake was calculated. The effect of diet was calculated.

3) Safety evaluation

a. Adverse Events, b. Electrocardiogram, c. Diagnostic medical test, d. vital signs, e. Complication test: microalbuminuria (urine test) is performed only during screening.

* Subjects must visit the hospital with fasting for at least 8 h before collecting blood.

* Diagnostic medical test items (test results conducted within the past 4 weeks based on the random assignment date can be used).

■ Hematological test: WBC, RBC, Hemoglobin, Platelets counts

■ Blood biochemical tests: Albumin, Total protein, ALT, AST, r-GT, Alkaline phosphatase, Triglyceride, Total cholesterol, HDL cholesterol, LDL cholesterol, glucose, BUN, creatinine, uric acid tests are carried out on a fixed schedule.

■ Urine test: pH, Protein, Glucose, WBC, RBC, Specific gravity, albumin (microalbuminuria, only conducted at visit 1), HCG

■ Pregnancy test (only if there is a possibility of pregnancy among the subjects, check the pregnancy status at each visit)

4) Functional evaluation (diabetes-related indicator test): Fasting blood glucose, HbA1c, Insulin, and C-peptide are laboratory tests performed at each visit. OGTT (2 h) is performed only at Visits 2 and 4. If 8 h on an empty stomach has not passed, visit again, and perform a blood sugar test on an empty stomach.

5) Subject suitability evaluation: After each screening test, the results are reviewed and judged.

6) Follow-up visit: If it is deemed necessary to follow up after the final administration of the test food or after early termination at the discretion of the tester, abnormal laboratory results, continuing adverse reactions, dropout, and follow-up according to the tester's discretion. You should come back within 4 weeks for this.

* Elimination Criteria: All prospective subjects who performed screening should be listed in the subject log even if they are excluded from the selection of subjects.

**Table S4** Effect of CLM on lowering HbA1c over subjects with decreased levels of FBG, insulin, C-peptide, insulin, HOMA-IR, and HOMA-IR by C-peptide.

| **Evaluation variables** |  | **Placebo** | **CLM** | **χ2** |
| --- | --- | --- | --- | --- |
| Total subjects | Total subjects | 36 | 36 | 0.237 |
|  | Subjects with decreased HbA1c | 13 | 18 |  |
| Subjects with decreased FBG | Total subjects | 16 | 21 | 0.025748 |
|  | Subjects with decreased HbA1c | 2 | 10 |  |
| Subjects with decreased insulin | Total subjects | 15 | 24 | 0.037237 |
|  | Subjects with decreased HbA1c | 3 | 13 |  |
| Subjects with decreased C-peptide | Total subjects | 17 | 25 | 0.068302 |
|  | Subjects with decreased HbA1c | 4 | 13 |  |
| Subjects with decreased HOMA-IR | Total subjects | 15 | 24 | 0.037237 |
|  | Subjects with decreased HbA1c | 3 | 13 |  |
| Subjects with decreased HOMA-IR by C-peptide | Total subjects | 19 | 25 | 0.038978 |
|  | Subjects with decreased HbA1c | 4 | 13 |  |

**Table S5** Lipid changes at the baseline and after 12 weeks

| **Evaluation variable** |  | **Control** | **Test group** | ***p* value^a^** | ***p* value^b^** |
| --- | --- | --- | --- | --- | --- |
|  |  | **Mean±SD** | |  |  |
| Total Cholesterol | Baseline | 187.2±32.9 | 196.0±27.4 | 0.431 |  |
|  | after 12 weeks | 191.9±29.5 | 197.0±24.2 | 0.421 |  |
|  | Difference^c^ | -3.7±30.2 | -3.0±28.1 | 0.942 |  |
|  | *p* value^d^ | 0.831 | 0.905 |  | 0.554 |
| TG | Baseline | 129.0±62.9 | 132.8±58.2 | 0.842 |  |
|  | after 12 weeks | 129.8±58.6 | 144.4±87.0 | 0.409 |  |
|  | Difference^c^ | 4.7±76.2 | -12.8±78.6 | 0.34 |  |
|  | *p* value^d^ | 0.753 | 0.921 |  | 0.317 |
| HDL | Baseline | 53.8±11.3 | 54.3±13.4 | 0.826 |  |
|  | after 12 weeks | 53.9±10.4 | 53.6±11.4 | 0.897 |  |
|  | Difference^c^ | -0.8±10.7 | 0.2±7.2 | 0.652 |  |
|  | *p* value^d^ | 0.843 | 0.541 |  | 0.626 |
| LDL | Baseline | 109.6±26.9 | 109.6±26.9 | 0.186 |  |
|  | after 12 weeks | 117.5±28.1 | 121.9±24.3 | 0.495 |  |
|  | Difference^c^ | -7.1±28.5 | -3.3±24.6 | 0.543 |  |
|  | *p* value^d^ | 0.758 | 0.771 |  | 0.897 |

a: Compared between groups: *p*-value by independent *t*-test

b: Compared between groups: *p*-value by ANCOVA (adjustment with baseline and calorie)

c: Difference between the values of baseline and after 12 weeks.

d: Compared within groups: *p*-value by paired *t*-test

**Table S6** Changes in vital signs

| **Evaluation variable** |  | **Control** | **Test group** | ***p* value^a^** | ***p* value^b^** |
| --- | --- | --- | --- | --- | --- |
|  |  | **Mean±SD** | |  |  |
| Systolic BP | Baseline | 122.7±10.9 | 121.6±10.5 | 0.66 |  |
|  | after 12 weeks | 122.8±10.4 | 121.1±10.5 | 0.497 |  |
|  | Difference^c^ | 0.7±7.7 | 0.1±14.4 | 0.829 |  |
|  | *p* value^d^ | 0.583 | 0.961 |  | 0.734 |
| Diastolic BP | Baseline | 75.4±8.6 | 73.5±8.6 | 0.34 |  |
|  | after 12 weeks | 75.4±7.5 | 72.8±6.6 | 0.141 |  |
|  | Difference^c^ | 0.4±6.7 | 0.0±7.7 | 0.83 |  |
|  | *p* value^d^ | 0.721 | 0.982 |  | 0.45 |
| Pulse | Baseline | 69.8±9.5 | 72.9±10.8 | 0.199 |  |
|  | after 12 weeks | 74.5±10.3 | 75.0±13.0 | 0.863 |  |
|  | Difference^c^ | -4.9±7.9 | -3.6±9.2 | 0.561 |  |
|  | *p* value^d^ | 0.001 | 0.033 |  | 0.641 |

a: Compared between groups: *p*-value by independent *t*-test

b: Compared between groups: *p*-value by ANCOVA (adjustment with baseline and calorie)

c: Difference between the values of baseline and after 12 weeks.

d: Compared within groups: *p*-value by paired *t*-test

**Table S7.** Changes in stability evaluation index (blood test)

| **Evaluation variable** |  | **Control** | **Test group** | ***p* value^a^** | ***p* value^b^** |
| --- | --- | --- | --- | --- | --- |
|  |  | **Mean±SD** | |  |  |
| WBC | Baseline | 6.48±1.48 | 6.49±1.75 | 0.966 |  |
|  | after 12 weeks | 6.56±1.99 | 6.79±2.49 | 0.679 |  |
|  | Difference^c^ | -0.19±1.76 | -0.40±1.59 | 0.621 |  |
|  | *p* value^d^ | 0.525 | 0.166 |  | 0.621 |
| RBC | Baseline | 4.58±0.38 | 4.61±0.46 | 0.76 |  |
|  | after 12 weeks | 4.53±0.37 | 4.57±0.42 | 0.671 |  |
|  | Difference^c^ | 0.04±0.25 | 0.05±0.22 | 0.875 |  |
|  | *p* value^d^ | 0.345 | 0.204 |  | 0.971 |
| Hemoglobin | Baseline | 13.96±1.31 | 14.28±1.45 | 0.339 |  |
|  | after 12 weeks | 13.89±1.29 | 14.22±1.31 | 0.311 |  |
|  | Difference^c^ | 0.05±0.61 | 0.19±0.61 | 0.372 |  |
|  | *p* value^d^ | 0.615 | 0.091 |  | 0.618 |
| Platelet | Baseline | 243.81±60.96 | 245.11±56.44 | 0.925 |  |
|  | after 12 weeks | 237.68±57.04 | 237.25±60.95 | 0.977 |  |
|  | Difference^c^ | 7.56±28.99 | 2.94±28.51 | 0.516 |  |
|  | *p* value ^d^ | 0.138 | 0.564 |  | 0.561 |

a: Compared between groups: *p*-value by independent *t*-test

b: Compared between groups: *p*-value by ANCOVA (adjustment with baseline and calorie)

c: Difference between the values of baseline and after 12 weeks.

d: Compared within groups: *p*-value by paired *t*-test

**Table S8.** Changes in stability evaluation index (biochemical test)

| **Evaluation variable** |  | **Control** | **Test group** | ***p* value^a^** | ***p* value^b^** |
| --- | --- | --- | --- | --- | --- |
|  |  | **Mean±SD** | |  |  |
| Total Protein | Baseline | 7.65±0.38 | 7.54±0.27 | 0.516 |  |
|  | after 12 weeks | 7.52±0.42 | 7.51±0.33 | 0.165 |  |
|  | Difference^c^ | 0.15±0.32 | 0.02±0.29 | 0.88 |  |
|  | *p* value^d^ | 0.011 | 0.634 |  | 0.247 |
| Albumin | Baseline | 4.39±0.18 | 4.36±0.16 | 0.456 |  |
|  | after 12 weeks | 4.32±0.20 | 4.36±0.15 | 0.383 |  |
|  | Difference^c^ | 0.08±0.18 | 0.00±0.17 | 0.075 |  |
|  | *p* value^d^ | 0.016 | 1 |  | 0.121 |
| AST | Baseline | 26.11±5.24 | 25.94±7.11 | 0.91 |  |
|  | after 12 weeks | 26.88±6.30 | 28.72±10.37 | 0.385 |  |
|  | Difference^c^ | -1.03±4.08 | -2.31±7.15 | 0.37 |  |
|  | *p* value^d^ | 0.151 | 0.077 |  | 0.372 |
| ALT | Baseline | 21.06±8.88 | 21.33±11.14 | 0.907 |  |
|  | after 12 weeks | 21.29±10.55 | 24.28±15.21 | 0.355 |  |
|  | Difference^c^ | -0.74±5.29 | -2.72±9.14 | 0.281 |  |
|  | *p* value^d^ | <0.001 | <0.001 |  | 0.295 |
| ALP | Baseline | 71.97±25.77 | 76.19±22.05 | 0.458 |  |
|  | after 12 weeks | 71.79±26.39 | 72.00±20.87 | 0.972 |  |
|  | Difference^c^ | 0.15±9.25 | 1.97±8.89 | 0.418 |  |
|  | *p* value^d^ | 0.927 | 0.22 |  | 0.452 |
| rGTP | Baseline | 32.36±25.79 | 30.97±18.76 | 0.795 |  |
|  | after 12 weeks | 30.15±17.92 | 41.47±65.95 | 0.339 |  |
|  | Difference^c^ | 1.65±15.36 | -11.13±58.11 | 0.221 |  |
|  | *p* value^d^ | 0.536 | 0.287 |  | 0.226 |
| BUN | Baseline | 15.06±3.46 | 15.08±3.69 | 0.974 |  |
|  | after 12 weeks | 14.74±3.71 | 15.19±3.43 | 0.61 |  |
|  | Difference^c^ | 0.32±4.15 | 0.13±4.72 | 0.856 |  |
|  | *p* value^d^ | 0.653 | 0.882 |  | 0.652 |
| Creatinine | Baseline | 0.73±0.13 | 0.77±0.18 | 0.267 |  |
|  | after 12 weeks | 0.72±0.13 | 0.77±0.15 | 0.161 |  |
|  | Difference^c^ | 0.00±0.07 | 0.01±0.08 | 0.607 |  |
|  | *p* value^d^ | 0.1 | 0.5 |  | 0.819 |
| Uric acid | Baseline | 5.22±1.22 | 5.35±1.44 | 0.679 |  |
|  | after 12 weeks | 5.16±1.30 | 5.29±1.25 | 0.663 |  |
|  | Difference^c^ | 0.03±0.82 | 0.14±0.71 | 0.548 |  |
|  | *p* value^d^ | 0.852 | 0.27 |  | 0.736 |

a: Compared between groups: *p*-value by independent *t*-test

b: Compared between groups: *p*-value by ANCOVA (adjustment with baseline and calorie)

c: Difference between the values of baseline and after 12 weeks.

d: Compared within groups: *p*-value by paired *t*-test
